# Effects of Heme Oxygenase-1 on c-Kit-Positive Cardiac Cells

**DOI:** 10.3390/ijms222413448

**Published:** 2021-12-15

**Authors:** Qianhong Li, Chandrashekhar Dasari, Ding Li, Asma Arshia, Ahmed Muaaz Umer, Mohamed Riad Abdelgawad Abouzid, Yiru Guo, Roberto Bolli

**Affiliations:** Institute of Molecular Cardiology, Division of Cardiovascular Medicine, Department of Medicine, University of Louisville, Louisville, KY 40202, USA; qianhong.li@louisville.edu (Q.L.); chandrashekhar.dasari@louisville.edu (C.D.); Ding.Li@nationwidechildrens.org (D.L.); fas227@uky.edu (A.A.); ahmedmuaaz.umer@louisville.edu (A.M.U.); mohamedriadabdelgawad.abouzid@louisville.edu (M.R.A.A.); yiru.guo@louisville.edu (Y.G.)

**Keywords:** heme oxygenase-1, Nrf2, Ec-SOD, DNA synthesis, c-kit-positive cardiac cells

## Abstract

Heme oxygenase-1 (HO-1) is one of the most powerful cytoprotective proteins known. The goal of this study was to explore the effects of HO-1 in c-kit-positive cardiac cells (CPCs). Lin^NEG^/c-kit^POS^ CPCs were isolated and expanded from wild-type (WT), HO-1 transgenic (TG), or HO-1 knockout (KO) mouse hearts. Compared with WT CPCs, cell proliferation was significantly increased in HO-1^TG^ CPCs and decreased in HO-1^KO^ CPCs. HO-1^TG^ CPCs also exhibited a marked increase in new DNA synthesis during the S-phase of cell division, not only under normoxia (21% O_2_) but after severe hypoxia (1% O_2_ for 16 h). These properties of HO-1^TG^ CPCs were associated with nuclear translocation (and thus activation) of Nrf2, a key transcription factor that regulates antioxidant genes, and increased protein expression of Ec-SOD, the only extracellular antioxidant enzyme. These data demonstrate that HO-1 upregulates Ec-SOD in CPCs and suggest that this occurs via activation of Nrf2, which thus is potentially involved in the crosstalk between two antioxidants, HO-1 in cytoplasm and Ec-SOD in extracellular matrix. Overexpression of HO-1 in CPCs may improve the survival and reparative ability of CPCs after transplantation and thus may have potential clinical application to increase efficacy of cell therapy.

## 1. Introduction

The adult mammal heart contains a population of cells that express c-kit, the stem cell factor receptor, but lack hematopoietic lineage markers, referred to as lin^NEG^/c-kit^POS^ cardiac cells (CPCs) [1,2,3,4]. Beginning in 2010, we have shown in numerous studies that CPCs do not engraft into the heart and do not differentiate into cardiac myocytes following transplantation [1,5,6,7,8,9,10,11,12,13,14,15,16,17]. Despite this, many preclinical studies have demonstrated that administration of CPCs to animals with ischemic heart failure can produce an improvement in left ventricular (LV) performance and remodeling [1,5,6,7,8,9,10,11,12,13,14,15,16,17]. Although these results are encouraging, the therapeutic utility of CPCs is limited by the fact that the vast majority of transplanted CPCs die and/or fail to engraft shortly after their delivery into the damaged heart [1,9,10], so that, after 4–5 weeks, <3% of the cells initially present in the infarcted heart can be detected [1]. Therefore, identifying and developing new strategies to enhance CPC retention and survival following transplantation may help to maximize the therapeutic potential of these cells. 

Heme oxygenases (HO) are the rate-limiting step in the enzymatic degradation of heme to carbon monoxide (CO), bilirubin, and ferrous ions [18,19]. Of the three isoforms identified (HO-1, -2, and -3), the inducible isoform, HO-1, has emerged as a key player in the tissue defense against cellular injury in a large variety of systems, including the heart, with powerful anti-inflammatory, anti-apoptotic, and cytoprotective properties [18,19,20,21,22]. HO-1 appears to modulate numerous cellular characteristics that may influence the reparative capacity of CPCs. We have previously shown that preconditioning CPCs with the HO-1 inducer, cobalt protoporphyrin (CoPP), enhances cell survival in response to oxidative stress [23]. However, preconditioning CPCs with CoPP is not likely to be a clinically feasible strategy because of the possible off-target effects of this molecule on genes other than HO-1 and because the upregulation of HO-1 induced by CoPP is transient.

While HO-1 is considered one of the most powerful cytoprotective proteins in the endogenous antioxidant defense system [18,19], extracellular superoxide dismutase (Ec-SOD) is so far the only known antioxidant enzyme that functions to scavenge biologically toxic superoxide (O_2_^−^) in the extracellular space [24]. Nuclear factor-erythroid 2-related factor 2 (Nrf2) is a redox-sensitive transcription factor that maintains redox homeostasis by regulating antioxidant-response element (ARE)-dependent transcription and the expression of antioxidant enzymes, including Ec-SOD [25]. However, it is unknown whether HO-1 regulates Ec-SOD via the Nrf2 signaling pathway in CPCs. 

The major goal of this study was to determine the effects of either overexpression or the deletion of HO-1 on CPC proliferation and to gain insights into the underlying molecular mechanism. To comprehensively assess this issue, we isolated and expanded CPCs from wild-type (WT), HO-1 transgenic (TG), or HO-1 knockout (KO) mouse hearts. We determined the proliferation rate and the role of HO-1 in regulating Ec-SOD through the Nrf2 signaling pathway in these CPC populations. Furthermore, we compared the effects of HO-1 on DNA synthesis during the S-phase of CPC division in WT, HO-1^TG^, and HO-1^KO^ CPCs under normoxic (21% O_2_) and hypoxic (1% O_2_) conditions, the latter to mimic the microenvironment in cardiac scar tissue. We found that HO-1 upregulates Ec-SOD and activates Nrf2 in CPCs. Furthermore, we found that HO-1 promotes cell proliferation and new DNA synthesis during cell division not only in normoxic (21% O_2_) condition but also under severe hypoxic stress (1% O_2_). These actions of HO-1 should result in better CPC survival after transplantation and the improved efficacy of CPC-based cell therapy.

## 2. Results

### 2.1. Characterization of Murine WT, HO-1^TG^, and HO-1^KO^ CPCs

**Figure 1:**
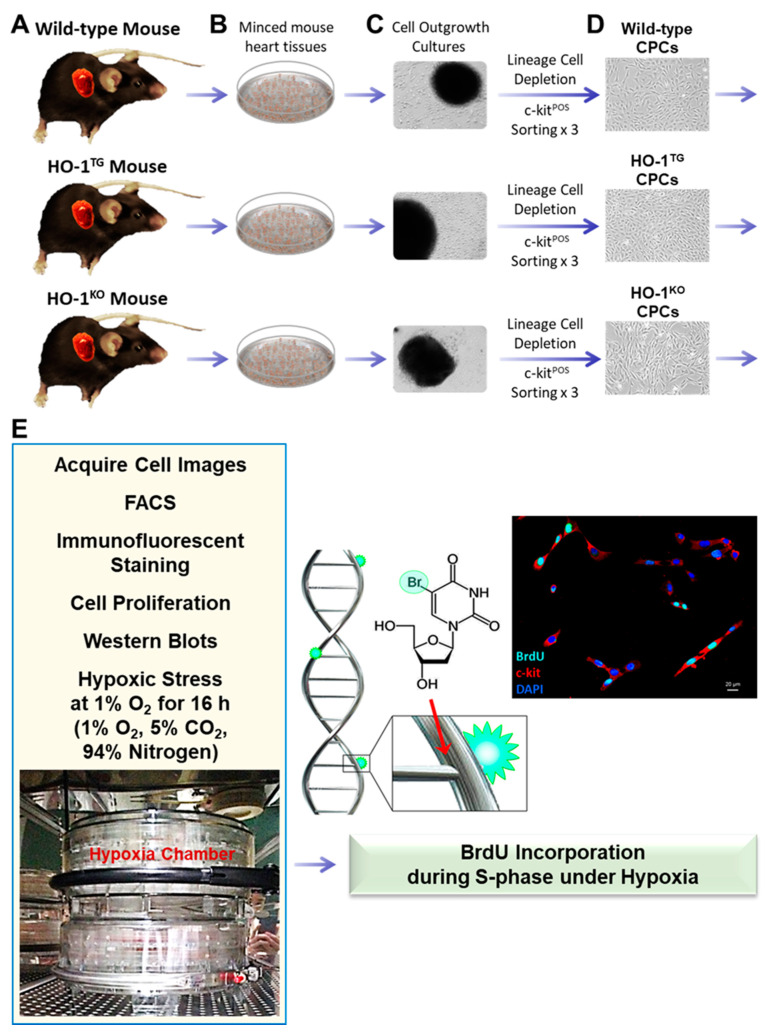
Overview of procedure. Mouse hearts were freshly isolated from WT, HO-1^TG^, or HO-1^KO^ mice (**A**), then minced (**B**) and cultured to establish cell outgrowth cultures over 7 days (**C**). The outgrowth cells were depleted of mature hematopoietic lineage positive cells and their committed bone marrow precursors with magnetic microbeads conjugated to a cocktail of antibodies against a panel of lineage antigens. The resulting cells were then sorted for c-kit with a specific anti-c-kit monoclonal antibody and magnetic immunobeads. To maximize purity, the c-kit sorting procedure was repeated three consecutive times at 14-day intervals (**D**). Six sets of endpoints (**E**) were used in this study, including cell image acquisitions, FACS, immunofluorescent staining, cell proliferation, Western blots and BrdU incorporation under hypoxia (1% O_2_ for 16 h).

Freshly minced hearts isolated from WT, HO-1^TG^, and HO-1^KO^ mice were cultured in 60 mm plates by the primary explant technique (Figure 1). After 7 days of primary culture, cell outgrowth was successfully obtained from all the hearts (*n* = 3/group). As shown in Figure 1, a monolayer of ~5000 cells was present around the periphery of the minced heart tissue. Lineage-positive cells were depleted as described in the Methods section of this report. The resulting unfractionated population was lin^NEG^ (54.5 ± 3.0% in WT, 54.6 ± 5.8% in HO-1^TG^, and 60.7 ± 6.6% in HO-1^KO^ CPCs; *n* = 3/group). Lin^NEG^ cells were further sorted with immunobeads against mouse c-kit to obtain an enriched population of lin^NEG^/c-kit^POS^ CPCs. 

**Figure 2:**
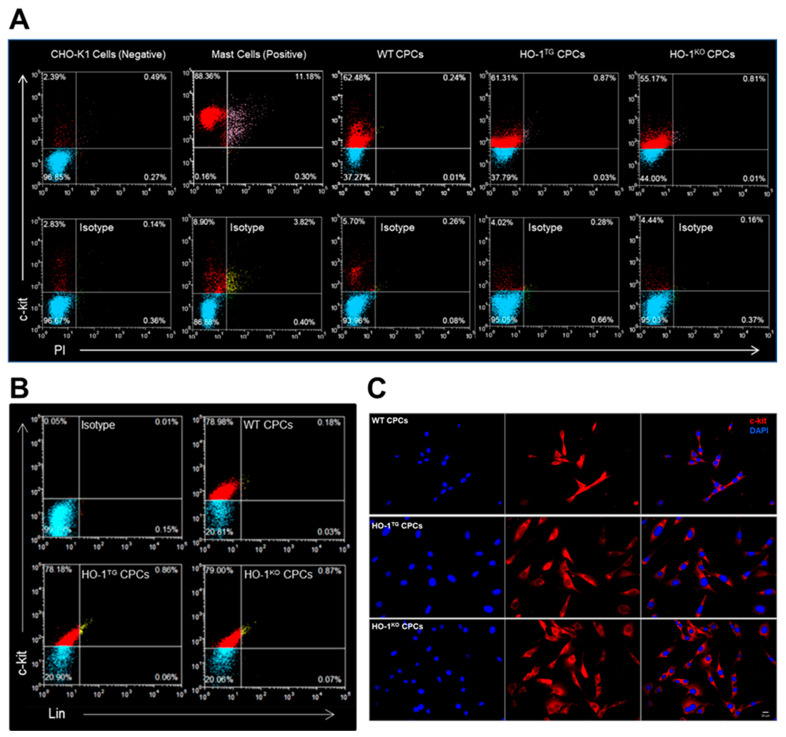
Characterization of WT, HO-1^TG^, and HO-1^KO^ CPCs. (**A**) Profile of live WT, HO-1^TG^, and HO-1^KO^ CPCs (passage 4) labeled with anti-mouse c-kit antibody (red), isotype control antibody (blue), and propidium iodide (PI, pink or yellow). Chinese hamster ovary cells (CHO-K1) were used as negative control and mast cells as positive control for c-kit FACS analysis. (**B**). Characterization of lineage negative (yellow) and c-kit positive (red) cells for WT, HO-1^TG^, and HO-1^KO^ CPCs (passage 4) by FACS. Cells labeled with isotype control antibody are shown in blue. (**C**). Live WT, HO-1^TG^, and HO-1^KO^ CPCs at passage 4 cultured in 35-mm glass bottom plates for 16 h were labeled with specific TR-conjugated anti-mouse c-kit antibody for 30 min, then fixed. Immunofluorescent staining showed WT, HO-1^TG^, and HO-1^KO^ CPCs expressing c-kit (red). Nuclei were stained with DAPI (blue). Bar: 20 µm.

The positivity for c-kit was determined in live CPCs by FACS. Propidium iodide (PI) was used to exclude possible false c-kit positive signals from cell debris and/or dead cells. As a negative control, c-kit positivity was 2.4% in Chinese hamster ovary (CHO-K1, American Type Culture Collection Inc., Manassas, VA, USA) live cells with 0.8% PI positivity. Although 99.9% c-kit positivity was observed in the positive control, the live mast cells (American Type Culture Collection Inc., Manassas, VA, USA), 11.5% of PI^POS^ cells should be excluded from mast cells due to false c-kit positive signals (Figure 2A). As illustrated in Figure 2A, c-kit positivity in live cells was 62.5% in WT, 61.3% in HO-1^TG^, and 55.2% in HO-1^KO^ CPCs with almost negligible PI positivity (0.3%, 0.9%, and 0.8%, respectively), indicating successful sorting of WT, HO-1^TG^, and HO-1^KO^ CPCs. Furthermore, live CPCs were fluorescently stained and labeled with lineage cocktail-biotin and anti-biotin-APC. In order to evaluate the c-kit positive fraction, the same live cells were additionally labeled with anti-mouse c-kit antibody-FITC and analyzed by flow cytometry. Figure 2B shows that a very weak lineage signal was detected in WT (0.2%), HO-1^TG^ (0.9%), and HO-1^KO^ (0.9%) CPCs, while a strongly positive c-kit signal was present in WT (79.0%), HO-1^TG^ (78.2%), and HO-1^KO^ (79.0%) CPCs. The expression of c-kit in the cell membrane was further confirmed by the immunofluorescent staining of WT, HO-1^TG^ and HO-1^KO^ CPCs cultured in 35-mm glass bottom plates (Figure 2C).

### 2.2. Effect of HO-1 on CPC Proliferation

**Figure 3:**
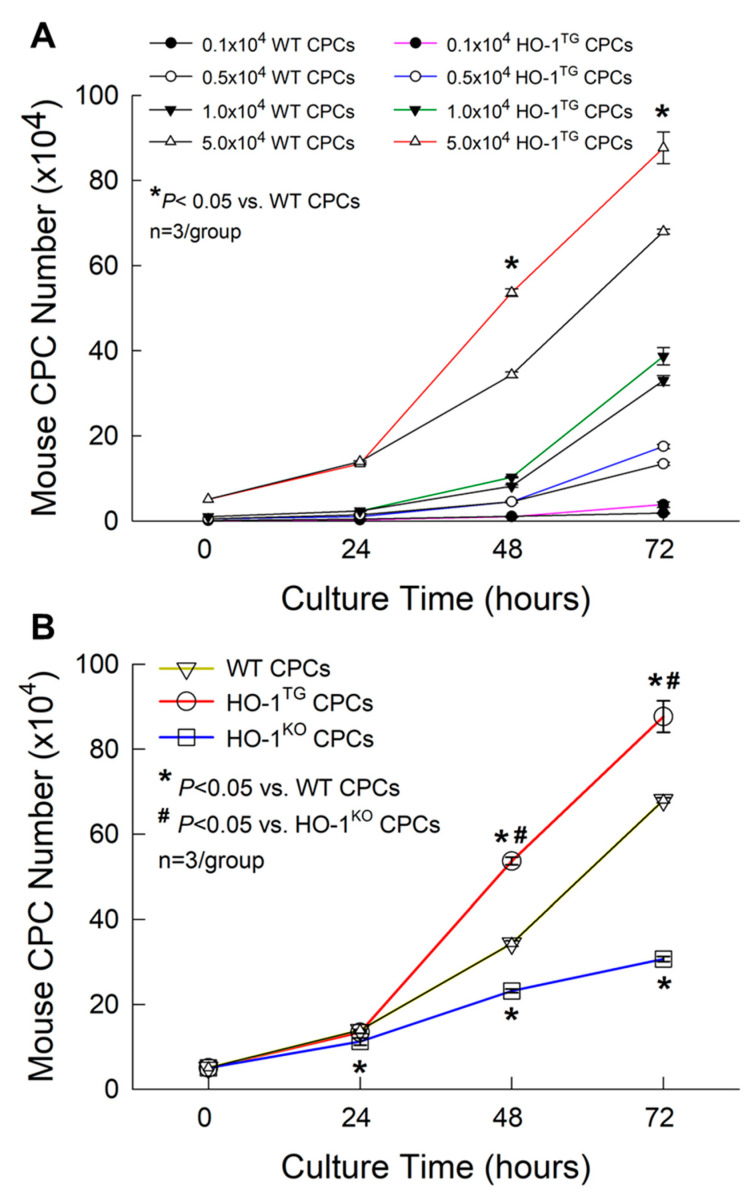
Effect of HO-1 on CPC proliferation. (**A**) Detection of the optimal cell density for assessing the effect of HO-1 on CPC proliferation. (**B**) WT, HO-1^TG^, and HO-1^KO^ CPCs were plated at a density of 5 × 10^4^ cells per well (culture area: 3.5 cm^2^) into 12-well plates. Note that after 48 h and 72 h of culture, the number of HO-1^TG^ CPCs was dramatically increased compared with WT and HO-1^KO^ CPCs. In contrast, deletion of the *HO-1* gene significantly reduced cell proliferation in HO-1^KO^ CPCs beginning at 24 h and up to 72 h of culture compared with WT CPCs. Data are mean ± SEM.

To optimize cell density for CPC proliferation studies, WT and HO-1^TG^ CPCs were plated at a density of 0.1 × 10^4^, 0.5 × 10^4^, 1 × 10^4^, and 5 × 10^4^ cells per well (culture area: 3.5 cm^2^) into 12-well plates. At 72 h of culture, regardless of cell density, cell proliferation was higher in the HO-1^TG^ group than in the WT group (Figure 3A). In particular, at a density of 5 × 10^4^ cells per well, overexpression of HO-1 significantly enhanced CPC proliferation at 48 h and up to 72 h of culture as compared with WT CPCs (*p* < 0.05, Figure 3A). Therefore, WT, HO-1^TG^, and HO-1^KO^ CPCs were plated at a density of 5 × 10^4^ cells per well and the number counted at 24 h, 48 h, and 72 h of culture. Compared with WT CPCs, deletion of the *HO-1* gene in CPCs resulted in a significant reduction in the number of HO-1^KO^ CPCs starting at 24 h and lasting until 72 h (*p* < 0.05, Figure 3B). In contrast, the overexpression of *HO-1* promoted CPC proliferation from 48 h to 72 h of culture as compared with the WT and HO-1^KO^ groups (*p* < 0.05, Figure 3B). 

Consequently, after only 3 days in culture, the total number of CPCs was ~1.3-fold and 2.8-fold greater in HO-1^TG^ CPCs than in WT and HO-1^KO^ CPCs, respectively (88 × 10^4^ vs. 68 × 10^4^ CPCs in the WT group and 31 × 10^4^ CPCs in the HO-1^KO^ group, *p* < 0.05; Figure 3B). When *HO-1* was overexpressed in CPCs, the CPC doubling time was reduced from 17.7 ± 0.8 h in the WT group to 16.2 ± 1.1 h in HO-1^TG^ group. In contrast, deletion of *HO-1* increased CPC doubling time from 17.7 ± 0.8 h in the WT group to 23.4 ± 2.2 h in the HO-1^KO^ group (*p* < 0.05). Thus, HO-1 not only plays an important role in maintaining basal CPC proliferation but also promotes a marked increase in CPC proliferation when overexpressed.

### 2.3. Effects of HO-1 on Nrf2 and Ec-SOD

**Figure 4:**
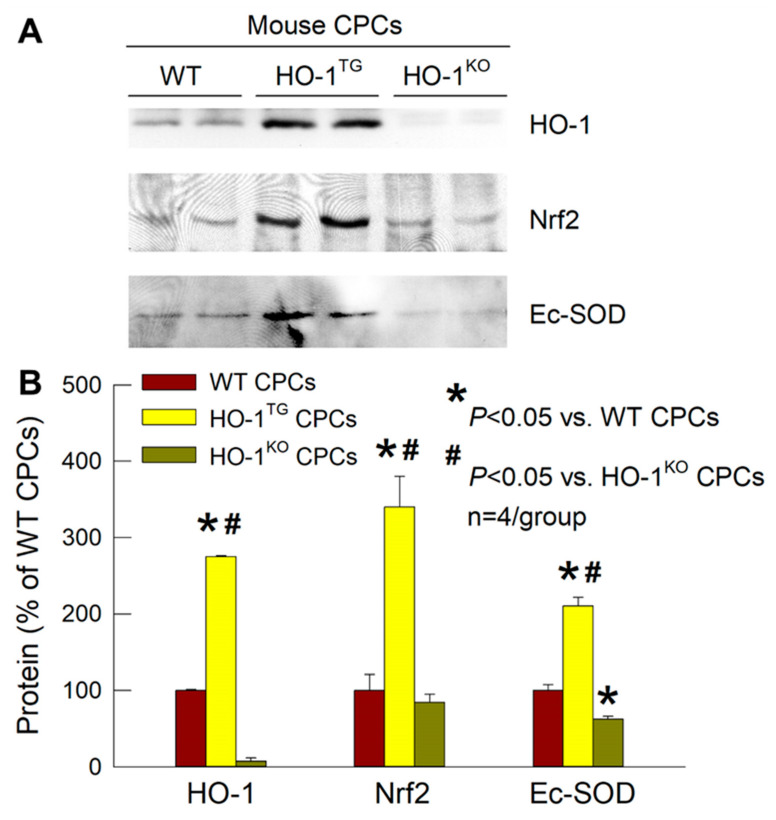
Effects of HO-1 on nuclear translocation of Nrf2 and expression of Ec-SOD. (**A**) Representative Western blots showing HO-1 overexpression in HO-1^TG^ CPCs and no detectable HO-1 in HO-1^KO^ CPCs (*top*), increased Nrf2 expression in the nuclear fraction in HO-1^TG^ CPCs but not in WT and HO-1^KO^ CPCs (*middle*), and increased Ec-SOD expression in HO-1^TG^ CPCs as compared with WT and HO-1^KO^ CPCs (*bottom*). (**B**) Quantitative analysis of HO-1, Nrf2, and Ec-SOD protein expression in WT, HO-1^TG^, and HO-1^KO^ CPCs. Data are mean ± SEM of experiments performed in duplicate (*n* = 4/group).

As expected, Western immunoblotting demonstrated HO-1 protein expression in HO-1^TG^ CPCs to be significantly increased (2.8-fold greater than WT CPCs; *p* < 0.05). In contrast, HO-1^KO^ CPCs had no HO-1 (Figure 4). To investigate the role of HO-1 in the resistance of CPCs to oxidative stress, we utilized HO-1^TG^ CPCs to overcome the relative lack of specificity of pharmacological HO-1 inducers that were reported previously [23]. As shown in Figure 4, the protein content of Nrf2 (a key transcription factor that regulates antioxidant genes in response to oxidative stress) in the nuclear fraction of HO-1^TG^ CPCs was increased 3.4-fold vs. WT CPCs and 4.0-fold vs. HO-1^KO^ CPCs (*p* < 0.05), indicating that HO-1 overexpression promotes translocation (and thus activation) of this transcription factor in the nucleus. At the same time, the protein expression of Ec-SOD was increased in HO-1^TG^ CPCs (+2.1-fold vs. WT CPCs and +3.4-fold vs. HO-1^KO^ CPCs; *p* < 0.05). In HO-1^KO^ CPCs, it was not only found that the level of nuclear Nrf2 was 10–15% lower than in WT CPCs but also the level of Ec-SOD protein expression was significantly reduced (40% lower than WT CPCs; *p* < 0.05, Figure 4). These results clearly demonstrate an important role of HO-1 in the regulation of Ec-SOD expression in CPCs and suggest that this occurs via the activation of the Nrf2 signaling pathway.

### 2.4. BrdU Incorporation into CPCs under Hypoxia

**Figure 5:**
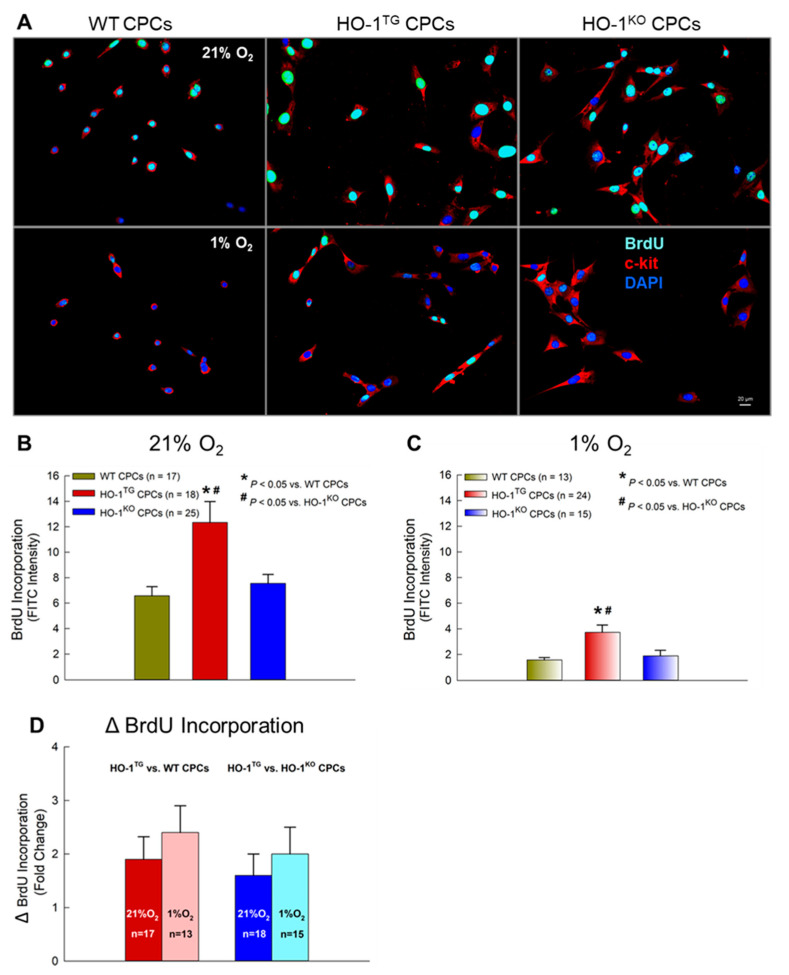
Effect of HO-1 on BrdU incorporation during S-phase of CPC division. (**A**) WT, HO-1^TG^, and HO-1^KO^ CPCs were cultured in 35-mm plates at 21% O_2_ for 24 h, then either at 21% O_2_ or at 1% O_2_ (hypoxic stress) for 16 h, and were subsequently treated with 10 µM BrdU for 30 min either at 21% O_2_ or at 1% O_2_ (hypoxic stress) culture condition. Immunofluorescent staining showed c-kit expression in red and BrdU incorporation in green/cyan. Nuclei were stained with DAPI (blue). Bar: 20 µm. (**B**,**C**) Quantitative analysis of BrdU incorporation under normoxia (21% O_2_) and hypoxic stress (1% O_2_). (**D**) Fold changes in BrdU incorporation in HO-1^TG^ vs. WT CPCs and in HO-1^TG^ vs. HO-1^KO^ CPCs under normoxia (21% O_2_) and hypoxic stress (1% O_2_) for 16 h. Data are mean ± SEM.

BrdU incorporation assays were used to assess the effect of HO-1 overexpression on new DNA synthesis during the S-phase of the cell cycle. A minimum BrdU concentration of 10 uM for 30 min was used. Under normoxic culture condition (21% O_2_), when BrdU was incubated with CPCs for 30 min at 10 uM, HO-1^TG^ CPCs exhibited an obvious increase in nuclear BrdU intensity (+1.9-fold vs. WT CPCs and +1.6-fold vs. HO-1^KO^ CPCs; *p* < 0.05, Figure 5A,B), indicating increased new DNA synthesis. Under hypoxic conditions (1% O_2_ for 16 h), a marked reduction in BrdU incorporation into the nuclei was observed in WT, HO^TG^, and HO-1^KO^ CPCs compared with normoxic conditions (Figure 5A–C). However, even in such a harsh culture environment, BrdU incorporation in HO-1^TG^ CPCs exhibited an increase (+2.4-fold vs. WT CPCs and +2.0-fold vs. HO-1^KO^ CPCs), which was actually greater than that seen under normoxic conditions (+1.9- and +1.6-fold, respectively) (Figure 5D). These results suggest that HO-1 enhances new DNA synthesis in both normoxic and hypoxic environments. 

## 3. Discussion

The purpose of this investigation was to determine, using a specific molecular transgenic or knockout approach, the effect of HO-1 on CPC proliferation and Ec-SOD content. To the best of our knowledge, this is the first demonstration that *HO-1* gene overexpression in CPCs enhances cell proliferation, upregulates Nrf2 and its downstream target, the antioxidant enzyme Ec-SOD, and promotes new DNA synthesis during the S-phase of cell division, not only in normoxia but also under hypoxic stress. Elucidating the effects of HO-1 on CPC proliferation and DNA synthesis may not only enhance and facilitate the culture of CPCs *in vitro* but also establish a new approach for potential clinical applications to increase the efficacy of cell therapy for heart failure *in vivo*.

The first working hypothesis in this study was that overexpression of the *HO-1* gene will increase DNA synthesis and CPC proliferation. The hypothesis was based on the following considerations. Although cell transplantation is emerging as a potentially transformative strategy to ameliorate LV remodeling and dysfunction after myocardial infarction, the beneficial effects observed in patients to date have been relatively modest [1,7]. Animal studies indicate that the vast majority (>95%) of transplanted cells die of apoptosis or vanish shortly after transplantation, and that the beneficial effects are produced by a very small minority of surviving cells [1,10]. These facts imply that manipulations on the transplanted cells that are cytoprotective may enhance the efficacy (and thus clinical applicability) of cell therapy for myocardial infarction and heart failure. One of the most powerful cytoprotective proteins currently known is HO-1 [19,26,27,28,29]. HO enzymes catalyze the degradation of heme (a pro-oxidant) to biliverdin, Fe^2+^, and carbon monoxide (CO), and biliverdin is rapidly converted to the powerful antioxidant, bilirubin [18]. HO-2 is constitutively active and widely distributed, whereas HO-1 is induced by a variety of cellular stresses and affords cellular protection in a variety of pathological cardiovascular conditions including myocardial infarction [19]. Our previous study used an inducer of HO-1, CoPP, to treat human CPCs *in vitro* at 10 µm for 24 h (concentrations > 10 µm were toxic to cells); this CoPP-preconditioning induced HO-1 protein expression in human CPCs which was associated with enhanced cell proliferation and survival *in vitro* and greater improvement of LV remodeling and cardiac function after transplantation in immunodeficient mice [30]. However, the use of pharmacological HO-1 inducers is severely limited by transient effects, potential toxicity, and relative lack of specificity. 

To overcome the above limitations, in this study we isolated, sorted and expanded CPCs from WT, HO-1^TG^, or HO-1^KO^ mouse hearts, an approach that enables us to study the effects of *HO-1* gene manipulations that are both specific and stable. WT, HO-1^TG^, and HO-1^KO^ CPCs were successfully isolated, sorted, and expanded, as demonstrated by the fact that they displayed stable morphology with high and consistent c-kit purity, lack of lineage markers, and no detectable changes in phenotype for up to 10 passages post-sorting (Figure 1 and Figure 2). We found that deletion of the *HO-1* gene resulted in a significant reduction in cell proliferation whereas the overexpression of *HO-1* promoted CPC proliferation above WT levels (Figure 3B). Thus, in addition to HO-1 being required to maintain basal CPC proliferation, HO-1 gain-of-function further increases CPC proliferation and thus could be used to reduce the time and cost needed to grow the large numbers of CPCs necessary for cell therapy.

The effects of HO-1 on CPC proliferation are further corroborated by our studies with BrdU, a synthetic thymidine analog that is incorporated into the new DNA strands of dividing cells during the S-phase of the cell cycle (when DNA replication occurs) [31]. BrdU was incubated with CPCs for only 30 min at a very low concentration (10 µM) [32]. Compared with WT CPCs, HO-1^TG^ CPCs exhibited a prominent increase in nuclear BrdU intensity under normoxic culture condition (21% O_2_) (Figure 5) as well as under the harsh hypoxic stress that we used (1% O_2_ for 16 h), when a marked reduction in BrdU incorporation was observed in WT, HO^TG^, and HO-1^KO^ CPCs compared with 21% O_2_ (Figure 5). These results indicate that HO-1 gain-of-function promotes new DNA synthesis in CPCs during the S-phase of cell division under both normoxic and hypoxic conditions, and suggest a cytoprotective effect in the latter.

The second working hypothesis in this study was that HO-1 regulates Ec-SOD in CPCs through the Nrf2 signaling pathway, which would afford protection against oxidative stress and likely enhance cell proliferation and survival *in vivo*. Among the members of the antioxidant enzymatic defense system, HO-1 is the most powerful antioxidant in the cytoplasm [33] whereas Ec-SOD is the only extracellular antioxidant [24,34,35,36]. Nrf2 is a master transcription factor that regulates important antioxidant genes [25,37,38,39]. In response to oxidative stress, Nrf2 translocates into the nucleus, where it binds to the antioxidant responsive element (ARE) to transactivate various genes encoding antioxidant enzymes [40]. The 5′-untranslated region of the *Ec-SOD* contains an ARE to which Nrf2 binds [41]. As illustrated in Figure 4, we found that HO-1 overexpression in CPCs was associated both with increased nuclear translocation (and thus activation) of Nrf2 and with increased Ec-SOD protein expression. When HO-1 was deleted via HO-1 knockout, the above upregulation of nuclear Nrf2 translocation and Ec-SOD protein expression disappeared, demonstrating that HO-1 upregulates Ec-SOD in CPCs and suggesting that this occurs via the activation of Nrf2. 

**Figure 6:**
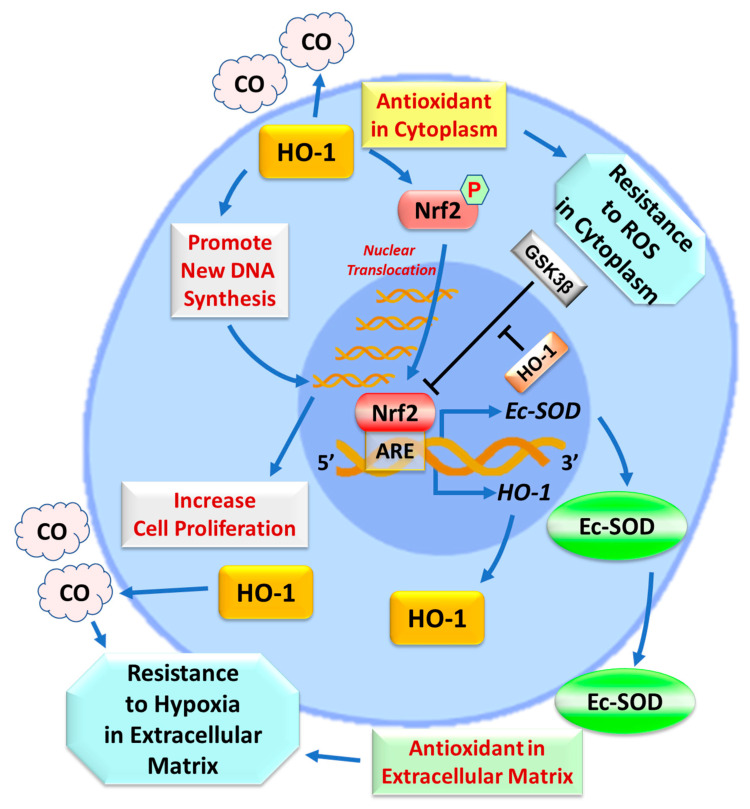
Effects of HO-1 overexpression on CPCs. HO-1 overexpression in CPCs promotes the phosphorylation and then the nuclear translocation of Nrf2, which binds to the ARE located on the 5′-untranslated regions of the *Ec-SOD* and *HO-1* genes and upregulates Ec-SOD and HO-1 expression. The nuclear HO-1, an enzymatically inactive isoform, can specifically facilitate the sustained stabilization of Nrf2 via the inhibition of GSK3β-mediated Nrf2 degradation so as to assist in the nuclear accumulation of Nrf2. Ec-SOD is the only antioxidant distributed in the extracellular matrix and surface of cells. Overexpression of HO-1 enhances new DNA synthesis during the S-phase of cell division so as to increase cell proliferation. As the most powerful antioxidant in the cytoplasm, HO-1 gain-of function can confer CPCs more resistance to stress. Additionally, the combination of upregulated Ec-SOD with CO, a highly diffusible gas produced by HO-1, can offer more antioxidant power to CPCs in the extracellular matrix and surface of cells as well as to adjacent cells that do not overexpress *HO-1* gene. These findings suggest that *HO-1* gene transduction may have salubrious effects in the clinical translation of cell therapy.

Although it is well known that HO-1 is positively regulated by Nrf2 (which leads to the mitigation of oxidative stress [19]), we have found that HO-1 overexpression via HO-1 transgenesis can upregulate the nuclear content of Nrf2, and that this phenomenon is abrogated in HO-1^KO^ CPCs. This is consistent with a previous study which showed that CoPP induced HO-1 overexpression in human CPCs, and that this was concurrent with the enhanced phosphorylation of Nrf2. Conversely, knockdown of Nrf2 by shRNA gene silencing eliminated the cytoprotective effects of CoPP, implying that they may involve the CoPP induced activation of an HO-1/Nrf2 axis [23]. It has been reported that nuclear HO-1, an enzymatically inactive isoform, can specifically interact with nuclear Nrf2 to facilitate Nrf2 sustained stabilization from GSK3β-mediated proteolytic degradation so as to promote nuclear accumulation of Nrf2 [42]. On the basis of our findings, we propose a novel mechanism of positive feedback in which HO-1 upregulates the activated Nrf2 in the nucleus, which in turn upregulates Ec-SOD and HO-1 expression, thus establishing an adaptive mechanism via the HO-1/Nrf2/Ec-SOD pathway in CPCs that enhances antioxidant defenses (Figure 6).

Our findings are consistent with the concept that HO-1, Nrf2, and Ec-SOD form a functionally-related and hierarchically-organized protective module in CPCs. We further propose that this module exerts cytoprotective actions, not only in the cytoplasm (HO-1) but also in the extracellular matrix, cell junctions and around the cell surface (Ec-SOD), thereby promoting CPC survival and proliferation. Additionally, HO-1 and Ec-SOD either produce a highly diffusible gas product (CO) [33] or are secreted into the extracellular space (Ec-SOD) [24]. The highly diffusible nature of CO and the extracellular location of Ec-SOD should enable these antioxidants to protect adjacent cells that do not express the *HO-1* transgene (including other cardiac cells), thereby producing a paracrine effect that would intercept reactive oxygen species (ROS) in the interstitial space and amplify the actions of HO-1-modified cell therapy *in vivo* (Figure 6). This unique property of Ec-SOD, coupled with the ability of CO to diffuse readily in tissues, suggests that the *HO-1* gene transduction of CPCs may have salubrious effects in the clinical translation of cell therapy that exceeds those strictly ascribable to CPC modification. 

In conclusion, we have shown that HO-1 gain-of function is beneficial to CPCs, increasing cell proliferation and DNA synthesis not only in normoxic but also in severe hypoxic conditions and promoting Nrf2 activation and Ec-SOD upregulation. These results reveal for the first time the impact of HO-1 on CPCs and may also apply to other types of cells used in cell therapy. So far, most preclinical and clinical studies of cell therapy for heart disease have used naïve cells without gene manipulation. Our data suggest that HO-1 transduction may be a potential strategy to enhance the therapeutic efficacy of CPCs for the treatment of heart failure. Additional studies will be needed to assess the effects of CPCs with HO-1 gain-of-function *in vivo* in a mouse model of myocardial infarction, in which extended retention after implantation of CPCs into the myocardium will be investigated.

## 4. Materials and Methods

### 4.1. Isolation of Murine CPCs

Murine lin^NEG^/c-kit^POS^ CPCs were isolated from adult WT, HO-1^TG^, or HO-1^KO^ mouse hearts (C57BL6, 10–12 weeks of age) and further purified by sorting [4]. HO-1^TG^ mice express human HO-1 cDNA under the control of the cardiac-specific mouse α-myosin heavy chain promoter [22]. HO-1^KO^ mice were generated by targeted disruption of the *HO-1* gene in embryonic stem cells [43]. Both the HO-1^TG^ and HO-1^KO^ mice were in the C57BL6 background. Briefly, mouse hearts were finely minced and cultured to establish cell outgrowth cultures over 7 days using growth medium (F12K medium supplemented with bFGF, LIF, and 10% FBS) [2,3,4]. Lin^NEG^/c-kit^POS^ CPCs were isolated from the cell outgrowth of the explants by sequential sorting. First, outgrowth cells were depleted of mature hematopoietic lin^POS^ cells, including T cells, B cells, thymocytes, monocytes/macrophages, granulocytes, neutrophils, erythrocytes, and their committed bone marrow precursors. For this purpose, cells were labeled with magnetic microbeads conjugated to a cocktail of antibodies against a panel of lineage antigens including CD5 (T and B lymphocytes and thymocytes), CD45R (B lymphocytes), CD11b (macrophages), GR-1 (granulocytes), 7–4 (neutrophils), and TER -119 (erythrocytes) (Miltenyi Biotec Inc., Auburn, CA, USA). This labeling procedure allows isolation of lineage negative cells lacking the markers of interest. The lin^NEG^ cells were then sorted for c-kit with a specific anti-c-kit monoclonal antibody (Santa Cruz Biotechnology, Inc., Dallas, TX, USA) and magnetic immunobeads (Miltenyi Biotec Inc., Auburn, CA, USA). To maximize purity, the c-kit sorting procedure was repeated three times at 14-day intervals (Figure 1). The purity of the sorted lin^NEG^/c-kit^POS^ cells was confirmed quantitatively by flow cytometry and immunofluorescent staining before use (Figure 2) [4]. In all experiments, CPCs were passaged less than 6 times.

### 4.2. Mouse CPC Culture

Mouse CPCs were cultured in growth medium [2,3,4] in a 21% O_2_ incubator supplied with 5% CO_2_ at 37 °C. Under a microscope, when cells reached ~70–80% confluence in the culture plate, cell images were acquired to monitor changes in cell morphology. Then, cells from one 100-mm culture plate were digested by trypsinization, counted with a hemocytometer under a microscope, split and seeded into new 100-mm culture plates at 5000 cells/cm^2^. A hypoxic chamber system containing 1% O_2_, 5% CO_2_, and 94% nitrogen was employed to mimic the microenvironment in cardiac scar tissue [44]. In all the experiments, after sorting, the mouse CPCs were passaged less than 6 times. 

### 4.3. Flow Cytometric Analysis

To verify the purity of the c-kit^POS^ cells in the sorted cell population, live CPC suspensions (1 × 10^6^ cells per aliquot) were labeled with specific anti-c-kit antibody and PI or lineage cocktail-biotin and anti-biotin-APC (Miltenyi Biotec Inc., Auburn, CA, USA). Samples were analyzed by flow cytometry (BD FACS Calibur and BD LSRII, Becton Dickinson, Franklin Lakes, NJ, USA), and 10,000 or 50,000 events were collected per sample (*n* = 4/group) [4].

### 4.4. Immunofluorescent Staining

To verify the purity of the c-kit^POS^ cells in the sorted cell population, WT, HO-1^TG^, or HO-1^KO^ CPCs were seeded at 5 × 10^4^ cells in 35-mm glass bottom plates for 16 h. Live CPCs in 35-mm plates were labeled with specific TR-conjugated anti-mouse-c-kit antibody (Miltenyi Biotec Inc., Auburn, CA, USA) for 30 min, then fixed in 4% paraformaldehyde for 15 min. After washing cells with PBS, the nuclei were stained with DAPI. Signals of c-kit positivity in red in the cytoplasm and membrane and DAPI in blue in nuclei were acquired with fluorescent microscopy [4]. 

### 4.5. Cell Proliferation

WT and HO-1^TG^ CPCs were plated at a density of 0.1 × 10^4^, 0.5 × 10^4^, 1 × 10^4^, and 5 × 10^4^ cells per well (culture area: 3.5 cm^2^) into 12-well plates to detect which cell density was optimal for the CPC proliferation study. Then, WT, HO-1^TG^, or HO-1^KO^ CPCs were seeded at 5 × 10^4^ cells per well into 12-well plates. After trypan blue staining, viable cell numbers were counted and compared at 24 h, 48 h, and 72 h of culture, and also used for the doubling time measurement. The experiments were independently performed in triplicate at each time-point in each group [44]. 

### 4.6. Calculation of Cell Doubling Time

The following formula was used to calculate the cell population doubling time (DT):

$$\mathrm{DT}=\frac{T\;\times \;\mathrm{Ig}2}{\mathrm{Ig}\;\left(\mathrm{Xe}\right)-\mathrm{lg}\left(\mathrm{Xb}\right)}$$

where T is the cell incubation time in any units, Xb is the cell number at the beginning of the incubation time, and Xe is the cell number at the end of the incubation time [45,46].

### 4.7. DNA Synthesis Assay

Cell proliferation was measured with the thymidine analog BrdU (5-bromo-2′-deoxyuridine) following its incorporation into newly synthesized DNA during the S-phase of mitosis through its subsequent detection with an anti-BrdU antibody. To assess the CPC proliferative ability in hypoxic condition, WT, HO-1^TG^, or HO-1^KO^ CPCs were cultured in 35 mm plates in a 21% O_2_ incubator supplied with 5% CO_2_ at 37 °C for 24 h. Then, they underwent either incubation at 21% O_2_ or hypoxic stress at 1% O_2_ for 16 h [47], and subsequently were treated with BrdU (10 µM) for 30 min at 21% O_2_ or 1% O_2_ [32]. Then, CPCs were fixed in 70% ethanol and stained with specific anti-BrdU and anti-mouse-c-kit antibodies (Miltenyi Biotec Inc., Auburn, CA, USA). The nuclei were stained with DAPI in blue. The BrdU nuclear positive signal in green/cyan occurs when a CPC in red is engaged in the process of new DNA synthesis. The intensity of the BrdU signal was assessed by fluorescent microscopy and quantitatively analyzed by Image J (1.48p, NIH) [31].

### 4.8. Western Immunoblotting Analysis

Protein samples were isolated from CPCs as previously described [21,48]. Nuclear protein fractions for detecting the nuclear Nrf2 were isolated from CPCs using the Nuclear Extract Kit (Active Motif, Carlsbad, CA, USA) according to the manufacturer’s instructions [48]. The expression of HO-1, Nrf2, or Ec-SOD in CPCs was assessed by standard SDS/PAGE Western immunoblotting with specific anti-HO-1, anti-Nrf2, and anti-Ec-SOD antibodies (Santa Cruz Biotechnology, Inc., Dallas, TX, USA) [20,21]. 

### 4.9. Statistical Analysis

The statistical methods were similar to those previously used by our group. Data are presented as means ± SEM. Data were analyzed with one-way analysis of variance (ANOVA for normally distributed data, or Kruskal–Wallis one-way analysis of variance on ranks for data that are not normally distributed, as appropriate), followed by unpaired Student’s *t* tests with the Bonferroni correction. A *p* value < 0.05 was considered statistically significant. All statistical analyses were performed using the SigmaStat software system (3.5V) [49,50,51,52,53].

## Data Availability

Not applicable.
